# Integrative multi-omics analysis identifies microbial dysbiosis and functional metabolic reprogramming in acute kidney injury

**DOI:** 10.3389/fmed.2026.1781145

**Published:** 2026-07-22

**Authors:** Guangli Fan, Ke Wang, Xin Qi, Yunxin Shi, Jialin Li, Yuanlong Zhang, Baoming Yang, Kexin Wang, Jizong Lv

**Affiliations:** 1 Universal Global Xi’an Beihuan Hospital, Xi’an, Shaanxi, China; 2 Tongji University School of Medicine, Shanghai, China; 3 Institute of Neuroscience, Air Force Medical University, Xi’an, Shaanxi, China

**Keywords:** acute kidney injury (AKI), gut microbiome, gut-kidney axis, metabolomics, multi-omics integration

## Abstract

**Background:**

Acute kidney injury (AKI) is a life-threatening syndrome with high morbidity and mortality, yet its early diagnosis and underlying mechanisms remain poorly defined. Emerging evidence implicates gut dysbiosis and microbial metabolic dysfunction in AKI pathogenesis via the gut-kidney axis, yet a comprehensive, multi-omics characterization of microbial functional alterations in general AKI populations remains lacking.

**Methods:**

We conducted a prospective multi-omics study including 16 patients with acute kidney injury (AKI) and 16 age- and sex-matched healthy controls (HCs). Plasma metabolomic profiling was performed using ultra-performance liquid chromatography coupled with quadrupole time-of-flight mass spectrometry (UPLC-QTOF/MS). Gut microbiome composition and function were characterized through whole-metagenome sequencing of stool samples. Differential taxonomic and metabolite features were identified using multivariate and univariate statistical analyses. Microbial functional potential was assessed across four hierarchical layers: Kyoto Encyclopedia of Genes and Genomes (KEGG) Orthologs (KOs) genes, pathways, gut-metabolite modules (GMMs), and gut-brain modules (GBMs), to achieve high-resolution mapping of metabolic pathways and taxon-specific functional contributions. Integrated microbe-metabolite-phenotype relationships were evaluated using Spearman correlation analysis.

**Results:**

Metabolomic profiling identified 65 differentially abundant metabolites between AKI patients and healthy controls (HCs), including 53 upregulated and 12 downregulated metabolites. These metabolites were mainly enriched in carbohydrate metabolism (e.g., starch and sucrose metabolism, fructose and mannose metabolism) and amino acid metabolism pathways. Among them, Maltol (C11918, AUC = 0.961), D-Quinovose (C02522, AUC = 0.926), and L-fucose (CO1019, AUC = 0.926) demonstrated the most robust diagnostic potential. Further feature selection using a random forest model identified an optimal panel of three metabolites, which achieved good discriminative performance (AUC = 0.859, 95% CI: 0.7073-1). Metagenomic analysis revealed significant gut microbiota dysbiosis in AKI, characterized by reduced α-diversity and distinct β-diversity compared to HCs. Taxonomic profiling showed depletion of key short-chain fatty acid-producing bacteria, including *Faecalibacterium prausnitzii*, along with enrichment of taxa such as *Phocaeicola* and *Bifidobacterium pseudocatenulatum*, as well as *Phocaeicola vulgatus* at the species level. Functional analysis indicated that AKI was associated with enhanced amino acid and carbohydrate metabolism, increased xenobiotic degradation, and alterations in neuroactive metabolic pathways. Integrated analysis further revealed significant correlations between altered microbial taxa, metabolic pathways, and clinical indicators. Specifically, health-associated taxa were negatively correlated with systemic inflammation markers (IL-6, IL-8) and renal injury markers (SCr, BUN), whereas *Bacteroides uniformis* showed positive associations with metabolic alterations in AKI.

**Conclusion:**

This multi-omics study reveals coordinated gut microbial dysbiosis and systemic metabolic reprogramming in AKI. The depletion of key commensals, rather than pathogen overgrowth, appears central to AKI-associated functional disruption. These findings highlight potential microbial and metabolic biomarkers and offer mechanistic insights into AKI pathogenesis.

## 1 Introduction

Acute kidney injury (AKI) is a critical clinical syndrome characterized by a rapid decline in renal function, carrying significant risks of morbidity, mortality, and progression to chronic kidney disease (CKD) (1, 2). Despite advances in supportive care, the underlying pathophysiological mechanisms of AKI remain incompletely understood, and early diagnosis continues to be a major clinical challenge due to the reliance on delayed markers such as serum creatinine (3). This diagnostic gap underscores the urgent need to identify novel, sensitive biomarkers and to elucidate the complex molecular networks driving kidney injury.

Recent advances in multi-omics technologies, including metabolomics, metagenomics, proteomics, and transcriptomics, have transformed the ability to dissect disease mechanisms at a systems level (4, 5). Metabolomics offers a functional readout of cellular activity and captures rapid biochemical perturbations associated with renal injury and repair (6, 7). Recent studies and reviews have highlighted that AKI is accompanied by extensive metabolic reprogramming, characterized by mitochondrial dysfunction, impaired oxidative phosphorylation, and compensatory activation of glycolysis, reflecting a profound bioenergetic imbalance in renal tubular cells (8, 9). These metabolic alterations are increasingly recognized as being closely associated with the progression and severity of kidney injury.

In parallel, growing evidence has emphasized the importance of the gut-kidney axis in AKI. Metagenomic and experimental studies have demonstrated that the gut microbiota plays a key role in regulating host metabolism, immune responses, and epithelial barrier integrity, thereby influencing susceptibility to kidney injury (10, 11). Recent literature further indicates that gut microbial dysbiosis-characterized by depletion of short-chain fatty acid (SCFA)-producing commensals and expansion of potentially pathogenic taxa-is associated with systemic inflammation, oxidative stress, and accumulation of uremic toxins across kidney diseases (12–15). These processes are thought to contribute to renal dysfunction and may influence the transition from AKI to CKD (16, 17). However, the specific microbial taxa and functional pathways involved in non-septic, general AKI populations remain insufficiently defined.

Although several studies have begun to integrate omics data in kidney injury research, most have focused on isolated etiologies, such as sepsis-associated AKI, ischemia-reperfusion injury, or drug-induced nephrotoxicity, or have been limited to a single omics layer (10, 18–20). For instance, multi-omics analyses in Sepsis-Associated Acute Kidney Injury (SA-AKI) have identified metabolite-microbe associations and pathway alterations (e.g., lysine degradation) specific to septic inflammation (5), while CKD studies have linked gut microbial dysbiosis with disruptions in circulating and fecal metabolomes (10). Recent integrative reviews have emphasized the need for simultaneous characterization of microbial functional capacity and host metabolic status to distinguish disease-specific alterations from generalized inflammatory responses (12, 14).

However, a comprehensive, simultaneous interrogation of gut microbial composition, microbial functional capacity, and systemic metabolic alterations in general AKI populations is notably lacking. Such integrative profiling is essential to distinguish AKI-specific changes from broader inflammatory responses and to identify clinically relevant biological signatures that transcend individual pathways or isolated etiologies (21).

Therefore, the present study employs an integrative multi-omics approach, combining metagenomics and metabolomics to systematically characterize gut microbial composition, functional pathways (including gut-metabolite modules and gut-brain modules), and plasma metabolic profiles in AKI. Through this approach, we aimed to provide a comprehensive overview of microbiome-metabolome interactions associated with AKI and to generate hypothesis-driven insights that may support the development of novel diagnostic biomarkers and microbiome-targeted therapeutic strategies.

## 2 Materials and methods

### 2.1 Study population

This prospective observational study was conducted from January to December 2024 at Universal Global Xi’an Beihuan Hospital. A total of 16 patients diagnosed with AKI and 16 age-and sex-matched HCs were initially enrolled. AKI was diagnosed according to the Kidney Disease: Improving Global Outcomes (KDIGO 2012) criteria, based on both serum creatinine (SCr) elevation and urine output parameters. All participants underwent screening following predefined inclusion and exclusion criteria. Exclusion criteria included chronic kidney disease, malignancy, autoimmune disease, chronic liver disease, recent infection, pregnancy, gastrointestinal disorders, or antibiotic or probiotic use within 4 weeks prior to recruitment. HCs were free of kidney disease, metabolic disorders, active infection, or recent medication use. Baseline demographic and clinical data, including renal function markers, liver enzymes, inflammatory indicators, and glucose metabolism-related indices, were systematically collected from electronic medical records. Biological samples, including blood and stool, were obtained at admission for subsequent multi-omics analyses.

To minimize potential confounding effects on gut microbiota and metabolomic profiles, participants with recent antibiotic, immunosuppressant, or probiotic use, as well as those unable to provide stool samples within 48 h of admission, were excluded. In addition, individuals with conditions known to substantially alter gut microbial composition or systemic metabolism were carefully excluded to further reduce microbiome-related variability. This study was designed to capture general features of AKI rather than focusing on a specific etiology. Therefore, patients were not stratified by underlying causes of AKI, with the aim of identifying shared microbiome-metabolome alterations across heterogeneous AKI conditions under controlled exclusion criteria. The primary objective of this study was to characterize alterations in the gut microbiome and plasma metabolome in AKI patients compared with healthy controls. Secondary analyses explored associations between microbial features, metabolic profiles, and clinical parameters.

The study protocol was approved by the Ethics Committee of Universal Global Xi’an Beihuan Hospital (Approval No. 20251220001). Written informed consent was obtained from all participants prior to enrollment.

### 2.2 Whole-metagenome shotgun sequencing and gut microbiome analysis

To characterize the gut microbial community, whole-metagenome shotgun sequencing was performed. Stool samples (≥500 mg) were collected using sterile scoops, rapidly frozen at −80 °C, and subsequently processed for microbial DNA extraction using the QIAamp DNA Stool Mini Kit (Qiagen, Germany). DNA integrity and concentration were assessed with a Qubit fluorometer and agarose gel electrophoresis. Metagenomic libraries were prepared from randomly sheared DNA fragments and sequenced as 150 bp paired-end reads on the MGI-SEQ2000 platform (MGI, Shenzhen, China).

Raw sequencing reads were quality-filtered using SOAPnuke (v1.5.2) and KneadData (v0.10.0) to remove low-quality bases, adapter contaminants. Host-derived sequences were removed using KneadData (v0.10.0) with Bowtie2 alignment against the human reference genome (hg38). Taxonomic profiling was performed using MetaPhlAn 4, which utilizes a comprehensive database of clade-specific marker genes derived from both reference genomes and metagenome-assembled genomes (MAGs), organized into species-level genome bins (SGBs). This approach enables accurate identification of both known and previously uncharacterized microbial species and provides improved taxonomic resolution compared with earlier versions. To enable broader detection of non-bacterial taxa-including archaea, viruses, and fungi-an additional classification step was conducted using Kraken2 (v2.1.2) against a comprehensive custom reference database. This database comprised 332,371 bacterial, 1,934 archaeal, and 18,639 viral genomes from NCBI RefSeq (accessed January 2024), along with 3,047 fungal genomes from NCBI RefSeq, FungiDB, and Ensembl Fungi (accessed July 2024). Taxonomic abundances were further refined using Bracken (v2.5.0). This combined strategy leverages the high specificity of MetaPhlAn 4 for bacterial profiling and the broad taxonomic coverage of Kraken2/Bracken, enabling comprehensive and robust characterization of the gut microbiome across multiple domains of life.

Functional potential of the gut microbiome was profiled using HUMAnN4 (v3.9) within the bioBakery suite, which aligns reads to UniRef90 protein families and reconstructs MetaCyc and KEGG pathway abundances. Differential microbial taxa were identified using the Wilcoxon rank-sum test at both phylum and genus levels, with *p* < 0.05 considered statistically significant. Linear discriminant analysis effect size (LEfSe) was applied to further characterize species-level biomarkers. Spearman correlation analysis was conducted to evaluate the relationships between microbial species abundance and host/functional features in AKI, including clinical parameters, gut metabolite modules (GMMs), gut-brain modules (GBMs), KEGG pathways, and KEGG orthologs (KOs). To correct for multiple testing, *p*-values were adjusted using the Benjamini-Hochberg false discovery rate (FDR) method. An adjusted *P* < 0.05 was considered statistically significant.

### 2.3 Untargeted metabolomic sequencing and analysis

Plasma was isolated from peripheral venous blood by centrifugation at 3,000 rpm for 10 min at 4 °C. Subsequently, 100 μL of plasma was transferred into a 1.5 mL EP tube and mixed with 400 μL of methanol. After vortexing, the mixture was incubated at −40 °C for 1 h to facilitate protein precipitation. Samples were then centrifuged, and 200 μL of the resulting supernatant was collected for metabolomic analysis. Chlorphenamine maleate was added as an internal standard for quality control. A pooled quality control (QC) sample was prepared by combining aliquots from all individual samples to monitor analytical stability. Metabolomic profiling was performed using ultra-performance liquid chromatography coupled with quadrupole time-of-flight mass spectrometry (UPLC-QTOF/MS; Thermo Fisher Scientific, USA).

Raw data were processed with Analyst software (v1.6.1, AB SCIEX, ON, Canada) for peak detection, retention-time alignment, and peak integration. Multivariate statistical analyses were conducted using SIMCA-P + (v15.0, Sartorius, Sweden). Principal component analysis (PCA) was first applied for unsupervised visualization and identification of potential outliers, followed by orthogonal partial least squares discriminant analysis (OPLS-DA) to enhance discrimination between AKI patients and HCs. Model robustness was evaluated through permutation testing and cross-validated R^2^Y and Q^2^ metrics. Metabolite identification was performed based on accurate mass matching and MS/MS (tandem mass spectrometry) spectral comparison against public databases, including HMDB and KEGG. According to the Metabolomics Standards Initiative (MSI) guidelines, this approach corresponds to Level 2 identification (putatively annotated compounds). To improve annotation confidence, only metabolites with high-quality MS/MS spectral matching (score ≥ 0.7) were retained for downstream analysis, while low-confidence features were excluded.

Differentially abundant metabolites were identified based on a combination of multivariate and univariate criteria: variable importance in projection (VIP) score ≥1.0 in the OPLS-DA model, combined with |fold change| ≥ 2 and *P* < 0.05 (two-tailed Student’s *t*-test). Functional enrichment analysis was performed using MetaboAnalyst (v5.0)^1^ to annotate differential metabolites and identify significantly enriched KEGG pathways, with significance determined by hypergeometric testing (*P* < 0.05). Receiver operating characteristic (ROC) curve analysis was conducted to evaluate the diagnostic performance of individual metabolites, and the corresponding area under the curve (AUC) values were calculated. In addition, a random forest model was applied for feature selection to identify an optimal subset of metabolites with the highest discriminative potential. Model performance was assessed using cross-validation to reduce the risk of overfitting. Given the relatively small sample size, the multivariate modeling results should be interpreted as exploratory. No independent external validation cohort was available, and further model optimization was intentionally limited to avoid overfitting and to maintain model robustness.

### 2.4 Statistical analysis

Statistical analyses were performed using SPSS (version 26.0) and R software (version 4.0). Comparisons between two groups were conducted using Student’s *t*-test.

## 3 Results

### 3.1 Clinical characteristics of AKI patients

In this study, a total of 32 participants were enrolled, including 16 patients with AKI and 16 HCs. The baseline demographic characteristics, including age, sex distribution, height, weight, and BMI, were comparable between the AKI and HCs groups, with no significant differences observed across these variables (all *p* > 0.05). In contrast, multiple clinical and biochemical parameters showed marked differences (Table 1).

**TABLE 1:** Patient characteristics.

Characteristic	AKI *N* = 16	HC *N* = 16	*p*-value
Demographics			
Gender (F/M)	11/5	9/7	0.465^1^
Age, year	34.69 ± 5.38	34.06 ± 5.98	0.758^2^
Height (cm)	165.19 ± 7.98	162.69 ± 8.43	0.396^2^
Weight (kg)	75.09 ± 8.16	71.81 ± 7.60	0.249^2^
Body mass index, BMI (kg/m2)	27.52 ± 2.41	27.10 ± 1.64	0.572^2^
Laboratory parameters Mean ± SD			
SCr (μmol/L)	244.64 ± 52.51	59.93 ± 5.11	<0.001^2^
BUN (mmol/L)	12.40 ± 2.97	3.73 ± 1.30	<0.001^2^
ALT (U/L)	168.73 ± 79.54	14.39 ± 3.43	<0.001^2^
AST (U/L)	139.74 ± 94.15	15.48 ± 2.18	<0.001^2^
GT (U/L)	218.51 ± 113.47	22.35 ± 11.27	<0.001^2^
ALP (U/L)	157.71 ± 67.47	86.93 ± 39.96	0.001^2^
Glu (mmol/L)	8.24 ± 1.72	5.24 ± 0.47	<0.001^2^
Urea (mmol/L)	11.86 ± 3.29	4.90 ± 0.69	<0.001^2^
WBC (10^9/L)	15.44 ± 2.28	9.64 ± 2.28	<0.001^2^
NLR	6.09 ± 3.38	6.39 ± 3.38	0.803^2^
IL_8 (pg/ml)	2,704.01 ± 2,402.38	152.52 ± 84.23	<0.001^2^
IL_1β (pg/ml)	168.21 ± 121.77	7.91 ± 2.83	<0.001^2^
IL_6 (pg/ml)	358.63 ± 229.97	50.59 ± 30.67	<0.001^2^
TNF_α (pg/ml) (IQR)	23.59 (10.08–55.93)	22.10 (17.56–26.07)	0.642^3^
IL_2 (pg/ml)	2,121.36 ± 1,111.82	1,731.83 ± 756.80	0.257^2^
IL_10 (pg/ml)	51.18 ± 36.52	13.44 ± 5.91	<0.001^2^
PCT (pg/ml)	52.75 ± 35.83	9.54 ± 6.75	<0.001^2^

^1^Pearson’s Chi-squared test;

^2^Welch Two Sample *t*-test;

^3^Wilcoxon rank sum exact test (non-normally distributed continuous variables); The data are presented as the mean ± SD. n.d., no data. *P*-values are calculated based on the Fisher’s exact test (gender) and Student’s *t*-test (other parameters).

Serum creatinine (SCr) and blood urea nitrogen (BUN) levels were significantly elevated in AKI patients compared with HCs (SCr: 244.64 ± 52.51 vs. 59.93 ± 5.11 μmol/L, *p* < 0.001; Blood Urea Nitrogen (BUN): 12.40 ± 2.97 vs. 3.73 ± 1.30 mmol/L, *p* < 0.001). Liver function markers, including Alanine Aminotransferase (ALT), Aspartate Aminotransferase (AST), Gamma-Glutamyl Transferase (GT), and Alkaline Phosphatase (ALP), were also significantly higher in AKI patients (all *p* ≤ 0.001). Similarly, fasting glucose and urea levels were elevated in AKI (*p* < 0.001). Regarding systemic inflammatory indices, White Blood Cell (WBC), IL-8, IL-1β, IL-6, and Procalcitonin (PCT) were markedly elevated in AKI patients (all *p* < 0.001). Notably, the anti-inflammatory cytokine IL-10 was significantly higher in AKI patients compared to HCs (51.18 ± 36.52 vs. 13.44 ± 5.91 pg/mL, *p* < 0.001). No significant differences were observed in Neutrophil to Lymphocyte Ratio (NLR) or IL-2 between groups. However, contrary to other pro-inflammatory markers, TNF-α showed no significant difference between the two groups (*p* = 0.642). Overall, these clinical profiles demonstrate that AKI patients show typical features of acute renal dysfunction accompanied by profound systemic inflammation and hepatocellular injury.

### 3.2 Whole-metagenome shotgun sequencing analysis results

**FIGURE 1:**
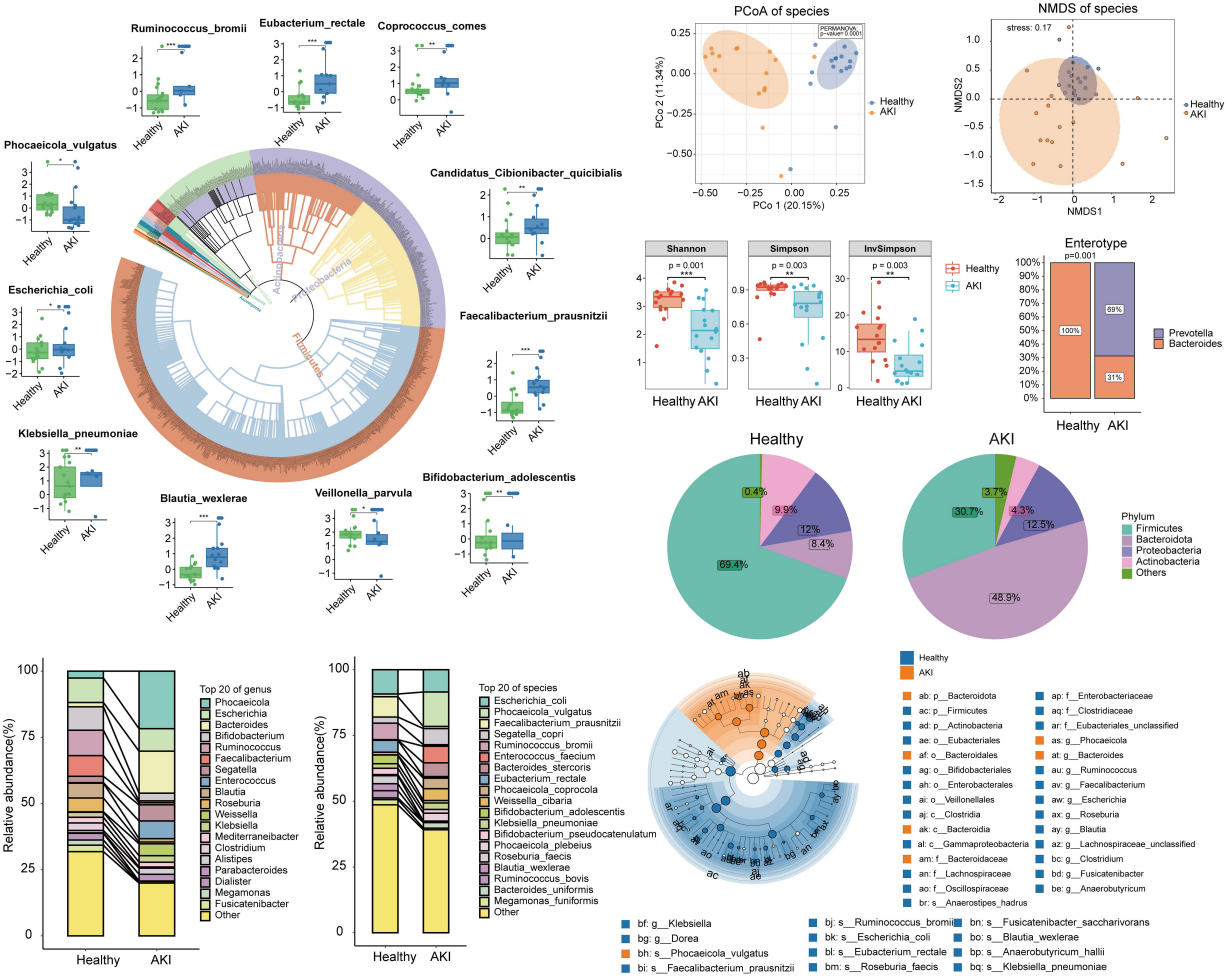
Taxonomic profiling and diversity analysis of gut microbiota in AKI patients: **(A)** Circular cladogram showing the phylogenetic relationships among microbial taxa at the phylum level, colored according to taxonomic classification. **(B,C)** Beta diversity analysis using PCoA and NMDS plots, based on Bray-Curtis dissimilarity, reveals distinct microbial communities between AKI and HCs. **(D)** Comparison of α-diversity indexes (Shannon, Simpson, and Inverse Simpson) of gut microbiota within HCs and AKI groups. **(E)** Enterotype analysis reveals altered enterotype distribution in AKI patients. **(F)** Pie charts presenting the relative abundance of microbial taxa at the phylum level in HCs and AKI groups. **(G,H)** Stacked bar diagram represent the relative abundance of top 20 dominant genus and species in the HCs and AKI groups. **(I)** The cladogram was obtained from the LEfSe analysis (LDA score > 2.0, *P* < 0.05). The colored circles from inside to out represent the classification level (phylum, class, order, family, and genus). The diameter of each small circle represents their abundance. Yellow nodes represent species with no significant difference and differential gut microflora are colored according to the group. The HC group was shown in blue, and the AKI group in red. *P < 0.05, **P < 0.01, ***P < 0.001.

To unravel the gut microbial signature of AKI, we performed fecal whole-metagenome shotgun sequencing on all participants (Supplementary Table 1). Taxonomic profiling identified 3 Kingdom, 13 phyla, 27 classes, 51 orders, 104 families, 328 genera, and 847 species (Figure 1A). The taxonomic tree illustrated clear compositional shifts between AKI patients and HCs. Then, we assessed and compared the microbiota diversity between groups. α-diversity was assessed using the Shannon, Simpson, and InvSimpson indices for the HC and AKI groups, results showed significantly reduced microbial diversity in the AKI group compared with HC group (*p* = 0.001, 0.003, 0.003, respectively, all *P* < 0.05) (Figure 1D), indicating a notable loss of microbial richness and evenness in AKI. β-diversity analyses further confirmed significant differences in microbial community structure between the two groups, Principal coordinates analysis (PCoA) demonstrated clear separation between AKI and HC groups (PERMANOVA *P* = 0.0001), while non-metric multidimensional scaling (NMDS) also revealed distinct clustering patterns with an acceptable stress value of 0.17 (Figures 1B, C), Together, these results indicate substantial shifts in overall microbial community structure in AKI patients. To further characterize the taxonomic differences between AKI patients and HCs, we compared microbial composition at multiple phylogenetic levels. Although both groups exhibited a *Prevotella*-dominant enterotype (Figure 1E), the *Prevotella*/*Bacteroides* ratio differed substantially, suggesting altered community balance and potential dysbiosis in AKI. At the phylum level, four major phyla-Firmicutes, Bacteroidota, Proteobacteria, and Actinobacteria-dominated the gut microbiota across all samples (Figure 1F). Specifically, Firmicutes showed a marked reduction in AKI (38.7%), whereas Bacteroidota displayed a relative increase (40.5%), indicating a potential imbalance in core microbial communities.

At the genus level, the top 20 most abundant genera exhibited distinct distribution patterns between groups (Figure 1G). Among them, 13 exhibited significant differences, including reductions in beneficial taxa such as *Anaerobutyricum, Fusicatenibacter, Faecalibacterium, Roseburia*, and *Blautia*, and increases in potentially pathogenic or opportunistic genera such as *Klebsiella, Escherichia, Phocaeicola*, and *Dialister*.

At the species level, the top 20 most abundant species were analyzed for differential abundance (Figure 1H). Of these, 11 species showed significant alterations between AKI and HCs, including decreases in beneficial commensals such as *Faecalibacterium prausnitzii, Eubacterium rectale, Bifidobacterium adolescentis, Bifidobacterium longum*, and *Roseburia faecis*, as well as increases in opportunistic or pathogenic taxa such as *Phocaeicola vulgatus*, and *Klebsiella pneumoniae.*

Additionlly, LEfSe analysis revealed pronounced microbial alterations between the AKI and HC groups. Taxa enriched in the AKI group were primarily located within the Bacteroidota lineage, including the class Bacteroidia, order Bacteroidales, family Bacteroidaceae, and *genera Phocaeicola* and *Bacteroides*. The species *Phocaeicola vulgatus* represented the most prominent AKI-enriched taxon, indicating a shift toward a Bacteroidota-dominated microbial community. Conversely, taxa enriched in healthy individuals were predominantly distributed within Firmicutes, particularly the class Clostridia and its downstream orders Eubacteriales and Oscillospirales. Enriched genera included *Faecalibacterium, Roseburia, Ruminococcus, Blautia*, and *Dorea*, accompanied by several health-associated, short-chain fatty acid-producing species such as *Faecalibacterium prausnitzii, Ruminococcus bromii, Eubacterium rectale, Roseburia faecis*, and *Blautia wexlerae.* These taxa collectively reflect a stable, metabolically beneficial microbial ecosystem in the healthy gut. Overall, the cladogram highlights a marked transition from a Firmicutes-enriched, SCFA-producing community in healthy controls toward a microbiota dominated by Bacteroidota lineages in AKI, indicative of disrupted microbial homeostasis associated with acute kidney injury (Figure 1I).

**FIGURE 2:**
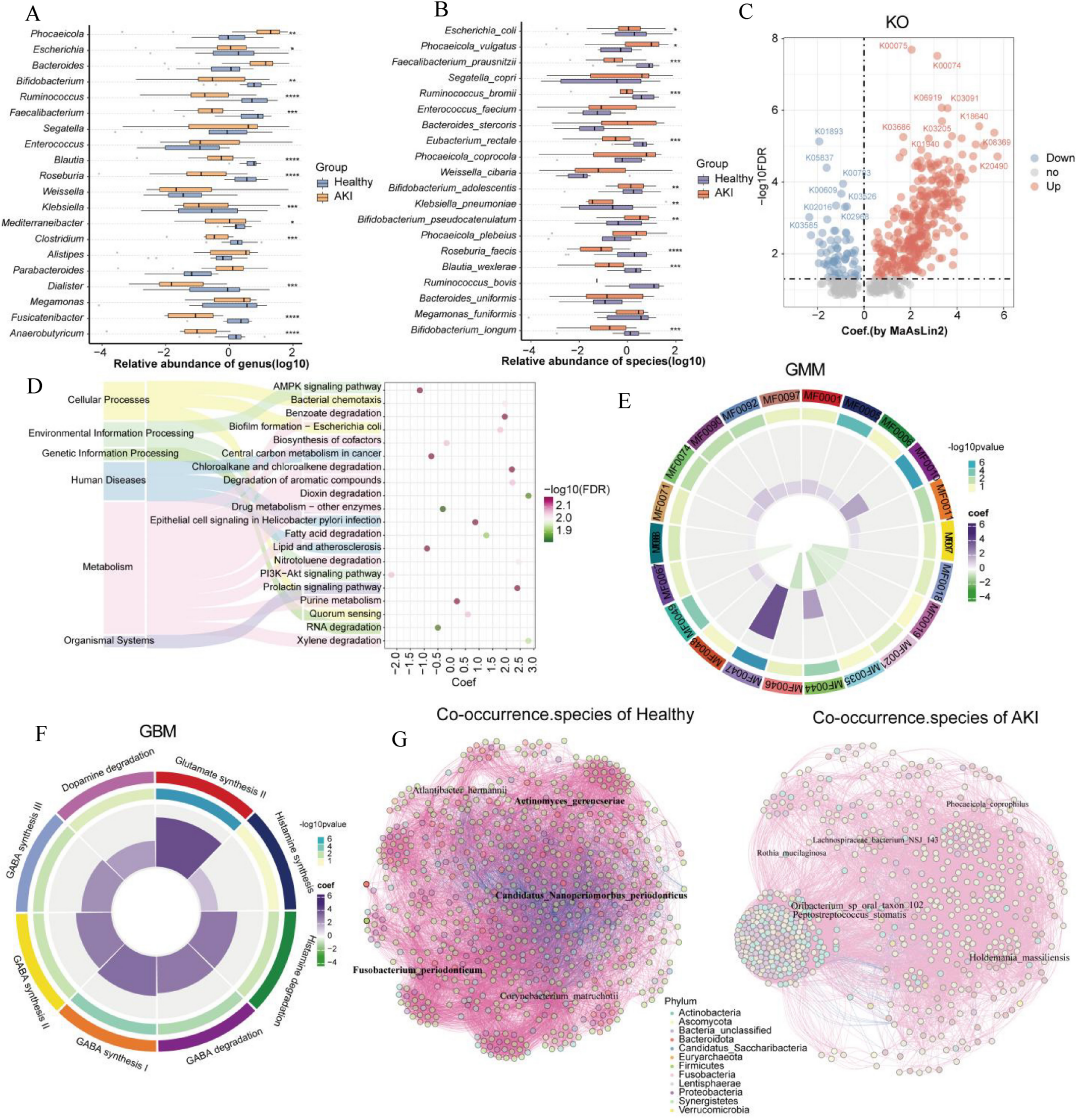
Multi-level functional profiling and host interaction analysis of gut microbiota in AKI based on metagenomic sequencing: **(A,B)** Box plots showing the Differential abundance of gut microbial taxa at the genus **(A)** and species **(B)** levels in AKI patients compared to HCs, with statistical significance indicated by *P < 0.05, **P < 0.01, ***P < 0.001, ****P < 0.0001. **(C)** Volcano plot of differentially enriched KEGG orthologs (KOs) between AKI patients and HCs. Blue points indicate down-regulated genes, red points indicate up-regulated genes, and gray points indicate genes with no significant differential expression. **(D)** KEGG pathway-level functional enrichment of altered microbial genes in AKI patients. Bubble size indicates the number of genes mapped per pathway, and color denotes enrichment significance, **(D)** KEGG pathway-level functional enrichment of altered microbial genes. Bubble color indicates directionality: red represent pathways enriched in AKI (positive coefficients), whereas green represent pathways enriched in healthy controls (negative coefficients). Bubble size indicates the number of genes mapped to each pathway, and color intensity reflects statistical significance (-log10 FDR). **(E,F)** Differentially enriched gut-brain modules (GBMs) and gut-metabolite modules (GMMs) between AKI and HCs. Bar plots show modules significantly enriched or depleted in AKI compared to HCs. Each bar represents a specific module, with bar length indicating the effect size (regression coefficient) and color denoting the statistical significance. **(G)** Co-occurrence networks of gut microbiota in HCs (left) and AKI patients (right). Nodes represent bacterial species; edges indicate significant positive correlations.

These findings indicate that while alpha diversity was significantly reduced in AKI, beta diversity analyses revealed clear separation between groups, suggesting substantial alterations in overall microbial community structure. To further elucidate the microbial alterations associated with AKI, we examined the top 20 most differentially abundant taxa at both the genus and species levels. At the genus level (Figure 2A), *Phocaeicola* emerged as the only taxon significantly enriched in AKI (*p* < 0.01). Several other Bacteroidota-associated genera, including *Alistipes, Parabacteroides, Segatella*, and *Bacteroides*, displayed higher median abundances in AKI; however, these differences did not reach statistical significance after multiple comparison correction, suggesting that the genus-level shifts were modest and not uniformly consistent across individuals. At the species level (Figure 2B), two taxa were significantly enriched in AKI: *Bifidobacterium pseudocatenulatum* (*p* < 0.01) and *Phocaeicola vulgatus* (*p* < 0.05). Both species displayed clear and reproducible increases in their median relative abundances in AKI compared with HCs. In addition to these significant findings, several other species displayed consistent upward trends in AKI, including *Segatella copri, Phocaeicola plebeius, Phocaeicola coprocola*, and *Enterococcus faecium*, although these changes did not reach statistical significance. The parallel elevation of multiple *Phocaeicola* species points toward a potential coordinated expansion of this lineage in AKI, even if individual species did not achieve sufficient effect size or statistical power to meet significance thresholds. Conversely, several beneficial short-chain fatty acid producing commensals, including *Faecalibacterium prausnitzii* and *Roseburia faecis*, tended to decrease in AKI, although these reductions were not statistically significant. Overall, these findings demonstrate that among the most prevalent gut taxa, *Phocaeicola* at the genus level and *Bifidobacterium pseudocatenulatum* and *Phocaeicola vulgatus* at the species level represent the most robust and significant discriminators between AKI and HCs, underscoring their potential relevance as microbial biomarkers of AKI-associated dysbiosis.

To explore the potential mechanisms underlying the associations between gut microbial features and AKI, we analyzed the microbial function alterations in Kyoto Encyclopedia of Genes and Genomes (KEGG) orthology (KO) genes, pathways, gut-brain modules (GBM, characterizing the neuroactive potential of the gut microbiota) and gut-metabolite modules (GMM, representing the metabolism of gut microbiota) in AKI patients compared to HCs. We found 492 differential KO genes between groups, among which 364 were increased and 128 were decreased in AKI patients compared with HCs (Figure 2C). The top 10 differential KO genes between groups included K00075 (murB), K00074 (paaH), K06919 (helicase), K03091 (sigH), K03205 (virD4), K18640 (parM), K08369 (ydjE), K02027 (ABC.MS.S), K03686 (dnaJ), K07816 (E2.7.6.5).

Kyoto Encyclopedia of Genes and Genomes functional profiling revealed marked metabolic differences between AKI patients and HCs (Figure 2D). In the AKI group, pathways involved in xenobiotic and aromatic compound degradation, including dioxin degradation, xylene degradation, nitrotoluene degradation, and chloroalkane and chloroalkene degradation, were significantly enriched, indicating enhanced microbial capacity for processing environmental pollutants. Quorum sensing was also elevated, suggesting strengthened microbial communication within the AKI gut ecosystem. Moreover, AKI samples showed enrichment of prolactin signaling and epithelial cell signaling in Helicobacter pylori infection, pointing to potential interactions with host inflammatory pathways. In contrast, HCs exhibited higher activity in several regulatory and metabolic pathways, including the AMPK signaling pathway, PI3K-Akt signaling pathway, central carbon metabolism in cancer, and RNA degradation, reflecting preserved microbial functions related to energy homeostasis and RNA turnover in the healthy gut.

Consistent with these pathway-level differences, gut microbial module (GMM) analysis demonstrated significant functional activation in AKI (Figure 2E). Modules involved in amino acid degradation, such as MF0047 (glutamine degradation II), MF0044 (cysteine degradation I), and MF0049 (threonine degradation I), were markedly enriched in AKI (all *q* < 0.05), with glutamine degradation II showing the strongest effect. AKI samples also exhibited increased capacities for carbohydrate breakdown (e.g., sucrose and starch degradation) and short-chain metabolite production, including ethanol, lactate, and pyruvate: formate lyase activity. In contrast, no GMM was significantly enriched in HCs, indicating that the functional distinction arises primarily from metabolic activation in AKI rather than from the presence of unique beneficial functions in health.

Gut-brain module (GBM) profiling further revealed pronounced alterations in neuroactive metabolic potential in AKI (Figure 2F). MGB007 (Glutamate synthesis II) showed the highest enrichment, and all three pathways for MGB020, MGB021, MGB022 (GABA synthesis (I-III)), as well as MGB019 (GABA degradation), were significantly elevated. MGB009 (Histamine degradation) was also enriched. Together, these GBM results highlight a functionally reprogrammed gut microbiome in AKI that actively modulates neurochemical signaling-particularly along the glutamate-GABA axis-and may contribute to systemic neuroimmune dysregulation during acute kidney injury.

At the ecological level, co-occurrence network analysis revealed substantial topological reorganization of the gut microbiome in AKI (Figure 2G). Although the AKI network appeared visually more fragmented, quantitative metrics demonstrated markedly increased connectivity compared with healthy controls, including higher edge count (35,198 vs. 13,281), average degree (113.4 vs. 41.2), clustering coefficient (0.896 vs. 0.439), and network density (0.183 vs. 0.064).

Importantly, centrality analysis further identified distinct hub taxa underlying network organization in each group. In the HC network, the top nodes ranked by degree included *Fusobacterium periodonticum, Actinomyces gerencseriae, Corynebacterium matruchotii, Atlantibacter hermannii*, and *Candidatus Nanoperiomorbus periodonticus*, forming a relatively distributed and balanced core structure. In contrast, the AKI network was dominated by a different set of highly connected taxa, including *Phocaeicola coprophilus, Rothia mucilaginosa, Lachnospiraceae bacterium NSJ-143*, and *Holdemania massiliensis*, indicating a shift toward alternative ecological hubs.

This reorganization was accompanied by reduced modularity (0.382 vs. 0.433) and increased degree centralization (0.203 vs. 0.101), suggesting a transition from a modular and resilient community to a more centralized, hub-dependent architecture. Collectively, these features indicate that the AKI gut microbiome is structured around a densely interconnected but less modular core, which may confer increased susceptibility to external perturbations and reduced ecological stability.

**FIGURE 3:**
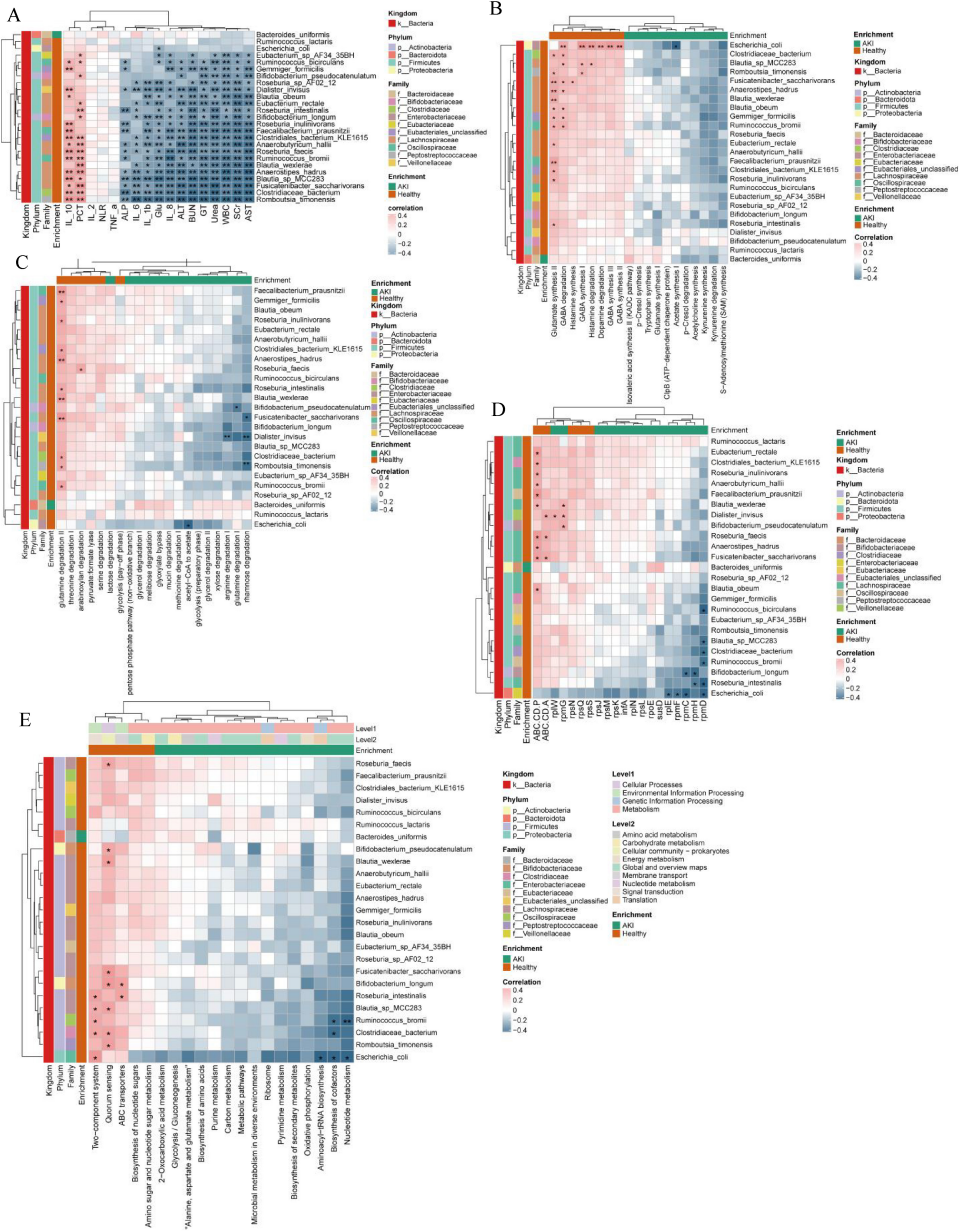
Heatmaps of Spearman correlations between differentially abundant gut microbial species and host/functional features in AKI. **(A)** Clinical parameters; **(B)** gut-metabolite modules (GMMs); **(C)** gut-brain modules (GBMs); **(D)** KEGG orthologs (KOs); **(E)** KEGG pathways. Color scale represents correlation coefficients, with red indicating positive correlations and blue indicating negative correlations. Asterisks denote statistical significance based on Benjamini–Hochberg false discovery rate (FDR)-adjusted *p*-values (*FDR < 0.05; **FDR < 0.01; ***FDR < 0.001).

To reveal the potential role of gut microbiota in the progression of AKI, we explored the associations between the differential abundant microbial species and phenotypes of AKI (Figure 3A). This correlation heatmap highlights a defining feature of gut dysbiosis in AKI: a broad depletion of health-associated commensals rather than an overgrowth of pathogenic species. Nearly all displayed taxa-including butyrate-producing *Faecalibacterium prausnitzii*, and immunomodulatory *Bifidobacterium pseudocatenulatum*, were enriched in HCs and exhibited significant protective correlations. These microbes showed strong negative associations with pro-inflammatory cytokines (IL-6, IL-8), systemic inflammation markers (WBC), renal injury indicators (Scr, BUN, urea), and liver enzymes (ALT, AST), while positively correlating with the anti-inflammatory cytokine IL-10. In sharp contrast, *Bacteroides uniformis* was the only taxon enriched in AKI, yet it showed no significant correlations with any clinical phenotype, suggesting that its enrichment may reflect a passive response to gut barrier disruption or metabolic shifts rather than direct involvement in inflammation or organ injury. Collectively, these findings underscore that AKI is characterized not by widespread pathogen expansion but by the collapse of a protective gut microbial network, which may contribute to uncontrolled inflammation and aggravated multi-organ dysfunction.

We further investigated the correlations between differentially enriched microbial taxa and host metabolic functions. Health-enriched taxa-such as *Faecalibacterium prausnitzii, Fusicatenibacter saccharivorans*, and *Roseburia intestinalis*-showed positive correlations with microbial regulatory pathways (two-component system, quorum sensing, ABC transporters) and negative correlations with AKI-associated metabolic pathways, including glycolysis, amino acid biosynthesis, and fatty acid biosynthesis. In contrast, the only AKI-enriched species, *Bacteroides uniformis*, showed no significant positive correlations with any KEGG pathways, indicating that it does not drive broad metabolic shifts at the pathway level (Figure 3E).

At the KO level, numerous ribosomal protein genes (e.g., rpmC, rpmH, rpmD) enriched in AKI exhibited predominantly negative correlations with health-associated taxa, suggesting that these KO increases are not attributable to the differential microbes identified. Conversely, several ABC transporter-related KOs (such as ABC.CD.P and ABC.CD.A) were positively correlated with health-enriched commensals. Notably, susD was the only KO positively associated with *B. uniformis*, representing the sole taxon-specific functional link in AKI (Figure 3D).

At the GMM level, multiple catabolic modules (ribose, arginine, and glutamine degradation) enriched in AKI and showed significant negative correlations with health-associated taxa such as *Fusicatenibacter saccharivorans, Bifidobacterium longum, Dialister invisus*, and *Clostridiaceae bacterium*, consistent with the loss of beneficial metabolic contributors. In contrast, glutamine degradation II was enriched in HCs and positively correlated with several health-associated species, including *Faecalibacterium prausnitzii, Gemmiger formicilis, Blautia obeum, Roseburia inulinivorans, Anaerostipes hadrus, Ruminococcus bicirculans, Bifidobacterium pseudocatenulatum*, and *Fusicatenibacter saccharivorans.* The only AKI-enriched species, *Bacteroides uniformis*, showed a significant positive correlation exclusively with glycerol degradation I, suggesting a narrow metabolic adaptation rather than broad functional activation (Figure 3C).

At the GBM level, several neuroactive and neuroprotective pathways, including GABA synthesis, dopamine degradation, histamine synthesis, and tryptophan synthesis, showed differential enrichment between groups, with distinct patterns observed in AKI and HCs (Figure 3B). Correlation analysis further revealed that these functional modules were associated with specific microbial taxa rather than directly reflecting their enrichment direction. In particular, beneficial commensals such as *Romboutsia timonensis, Roseburia inulinivorans, Roseburia intestinalis*, and *Gemmiger formicilis* were positively correlated with neuroactive pathways, whereas these taxa showed negative correlations with pathways such as S-adenosylmethionine (SAM) synthesis and kynurenine degradation. Notably, *Bacteroides uniformis* showed a specific positive association with isovaleric acid synthesis II, while exhibiting no consistent associations with other GBM modules, suggesting a limited and pathway-specific functional linkage.

Together, these multi-layered analyses reveal that AKI-associated dysbiosis is characterized not by a global gain of pathogenic functions, but by the collapse of symbiotic regulatory and metabolic networks-accompanied by the emergence of a narrowly adapted opportunist (*B. uniformis*) linked to specific detrimental activities, including polysaccharide scavenging (susD), glycerol utilization, and isovaleric acid production.

### 3.3 Metabolomic analysis results

**FIGURE 4:**
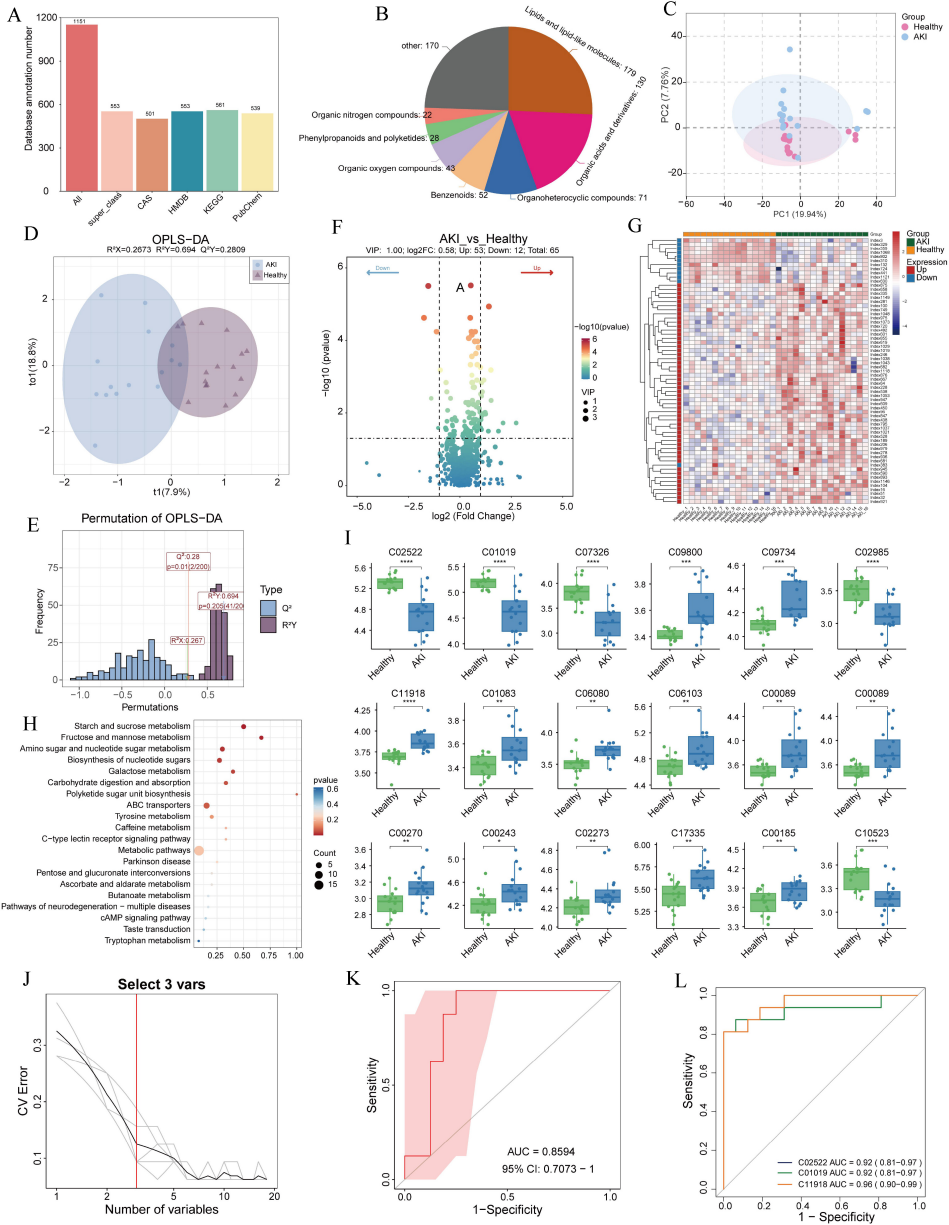
Metabolomic analysis between HCs and AKI patients: **(A)** Overview of metabolites identified in this study, categorized by their database sources (HMDB, KEGG, PubChem, etc.). **(B)** Classification and proportion of identified metabolites across major compound classes. **(C)** PCA scatter plots of metabolomes between HCs and AKI patients. **(D)** Orthogonal partial least squares discriminant analysis (OPLS-DA) score plot differentiating HCs and AKI patients. **(E)** Permutation test validating the OPLS-DA model with robust R2Y and Q2, values Q2 indicates model predictive ability; values of R2Y closer to 1 reflect better model fit. **(F)** Volcano plots of DAMs in HCs and AKI patients. The *x*-axis represents the log2-fold change in metabolites expression between the different groups. The *y*-axis represents the significance level of the expression difference. **(G)** Heatmap illustrating the relative abundance of all identified DAMs between HCs and AKI patients. Hierarchical clustering was applied to both samples and metabolites, with color intensity reflecting the standardized expression levels (red: up; blue: down). **(H)** KEGG Pathway Enrichment Analysis of DAMs. **(I)** The box plots shows the levels of 18 DAMs that are significantly altered in AKI patients compared to HCs. (*P < 0.05; **P < 0.01; ***P < 0.001; ****P < 0.0001). **(J)** Cross-validation error curve for random forest model optimization, with the red vertical line indicating the optimal number of variables (*n* = 3) where CV error reaches minimum. **(K)** Receiver operating characteristic (ROC) curve of the optimized random forest classifier with three metabolites (AUC = 0.859, 95% CI: 0.707–1.000). **(L)** Individual ROC analyses of the three selected metabolites showed that maltol (C11918) exhibited the strongest diagnostic performance (AUC = 0.96, 95% CI: 0.90–0.99), while D-quinovose (C02522) and L-fucose (C01019) demonstrated comparable discriminative ability (AUC = 0.92, 95% CI: 0.81–0.97).

To characterize the metabolome signatures of AKI, we performed untargeted mass spectrometry (MS) profiling of plasma polar metabolites in all AKI patients and HCs (Supplementary Table 2). After quality control of the metabolites measured in the blood, A total of 1,151 metabolites were successfully annotated across multiple databases (Figure 4A). Specifically, 553 metabolites were mapped to the superclass level, while 501, 553, 561, and 539 metabolites were annotated in the CAS, HMDB, KEGG, and PubChem databases, respectively, indicating broad metabolite coverage and high annotation confidence. This comprehensive annotation enables accurate downstream functional interpretation and pathway analysis. Class-level categorization further revealed that the identified metabolites spanned diverse chemical families (Figure 4B). Lipids and lipid-like molecules constituted the largest group (179), followed by organic acids and derivatives (*n* = 130) and organoheterocyclic compounds (*n* = 71). Additional categories included benzenoids (*n* = 52), organic oxygen compounds (*n* = 43), phenylpropanoids and polyketides (*n* = 28), and organic nitrogen compounds (*n* = 22). A remaining subset of 170 metabolites was assigned to other minor or unclassified categories. These results collectively demonstrate that the untargeted metabolomics approach captured a wide spectrum of metabolite classes, enabling a comprehensive biochemical overview of the metabolic alterations associated with AKI.

To assess the overall structure of the metabolomic data, principal component analysis (PCA) and orthogonal partial least squares discriminant analysis (OPLS-DA) were performed. As shown in Figure 4C, PCA indicated no clear separation between the AKI and HC groups, suggesting substantial inter-individual variability. In contrast, the OPLS-DA model showed a moderate level of group discrimination with R^2^Y = 0.694 and Q^2^Y = 0.2809 (Figures 4D, E). Although the predictive ability of the model was limited, permutation testing confirmed that the model was not overfitted. These results, together with subsequent univariate statistical analyses, suggest the presence of metabolic alterations associated with AKI. To identify differentially abundant metabolites (DAMs), variable importance in projection (VIP ≥ 1.0) value from the OPLS-DA model was combined with univariate statistical criteria (| fold change| ≥ 2 and *P* < 0.05), a total of 65 DAMs were identified between the AKI and HC groups, including 53 upregulated and 12 downregulated metabolites (Figure 4F). These findings suggest substantial metabolic alterations associated with AKI, providing potential insights into its underlying biochemical mechanisms. To further visualize the expression patterns of these DAMs, hierarchical clustering analysis was conducted. The resulting heatmap revealed two clearly separated clusters that corresponded to AKI and HC samples, demonstrating distinct and coordinated metabolic signatures between the two groups. Consistent upregulation of key metabolites in the AKI cluster and marked reductions in the HC cluster highlight the robust discriminatory power of these metabolic profiles (Figure 4G). Metabolic alterations can significantly influence associated biochemical pathways. To further explore these changes, KEGG pathway analysis was performed on the DAMs, The most significantly enriched pathways included starch and sucrose metabolism, fructose and mannose metabolism, amino sugar and nucleotide sugar metabolism, galactose metabolism, and carbohydrate digestion and absorption (all with *P* < 0.05) (Figure 4H). These findings suggest a profound disruption in carbohydrate metabolism during AKI. Additional enriched pathways involved tyrosine metabolism, tryptophan metabolism, ABC transporters, and cAMP signaling, indicating potential roles for amino acid metabolism, membrane transport, and cellular signaling in disease progression. Collectively, these enriched pathways demonstrate that AKI is characterized by extensive disruptions in carbohydrate, lipid, and amino acid metabolism, together with alterations in immune-related and neuroactive metabolic pathways, highlighting the complex biochemical reprogramming occurring during AKI. To identify metabolites with the highest biomarker potential, we focused on metabolites with VIP > 2 (*n* = 18). Boxplot analysis demonstrated that these metabolites exhibited clear and statistically significant differences in abundance between AKI patients and HCs (all *P* < 0.01, Figure 4I). Specifically, several carbohydrates, including D-quinovose (C02522) and L-fucose (C01019), were markedly downregulated, whereas metabolites such as farrerol (C09734) and maltol (C11918) were significantly upregulated, reflecting distinct alterations in carbohydrate-related and secondary metabolite pathways. To refine the biomarker panel, a random forest model was subsequently applied for feature selection. The cross-validation error curve indicated that the optimal model performance was achieved when the top three variables were retained (Figure 4J). Based on this minimal feature set, the combined diagnostic model demonstrated good discriminative ability, with an area under the curve (AUC) of 0.859 (95% CI: 0.7073–1) (Figure 4K). Notably, each of the three selected metabolites also exhibited strong individual predictive performance, with AUC values of 0.961, 0.926, and 0.926, respectively, underscoring their potential as robust biomarkers for distinguishing AKI from healthy states (Figure 4L).

## 4 Discussion

In this integrative multi-omics study, we characterized systemic metabolic remodeling and profound gut microbial dysbiosis in patients with AKI. Our findings demonstrate that AKI is not merely a localized renal insult but rather a systemic condition characterized by profound metabolic reprogramming and a distinct signature of gut microbial dysbiosis. Importantly, this dysbiosis appears to reflect not a generalized pathogen expansion, but a dual ecological pattern involving depletion of beneficial commensals and selective enrichment of opportunistic taxa, such as *Bacteroides uniformis*. This observation refines the current understanding of microbiome disruption in AKI. Recent systems-level studies of host-microbiome interactions have emphasized that disease-associated dysbiosis is often driven by ecological restructuring and functional imbalance, rather than simple overgrowth of pathogens, supporting the framework observed in our data.

Plasma metabolomic profiling identified 65 DAMs in AKI patients, with predominant alterations observed in carbohydrate metabolism (including starch/sucrose and fructose/mannose pathways) and amino acid metabolism. These findings indicate a marked systemic metabolic shift in AKI, particularly involving pathways related to energy production and substrate utilization. the enrichment of carbohydrate metabolism pathways may reflect a compensatory response to impaired mitochondrial function and reduced oxidative phosphorylation capacity. Under conditions of renal injury, tubular epithelial cells are known to undergo a bioenergetic transition characterized by decreased mitochondrial efficiency and increased reliance on glycolysis to sustain cellular energy demands. In this context, the metabolic patterns observed in our study likely represent a systemic manifestation of this energy imbalance, consistent with the concept of metabolic reprogramming in AKI (22–24). Emerging multi-omics evidence further suggests that this reprogramming extends beyond the kidney, involving coordinated alterations in redox homeostasis and substrate utilization, thereby supporting the view of AKI as a systemic metabolic disorder (8, 9). Building on this global metabolic shift, we further observed significant perturbations in amino acid pathways, particularly those involving tryptophan, tyrosine, and glutamine. These alterations have been associated with increased protein catabolism, systemic inflammation, and the accumulation of uremic toxins, including indole-and phenyl-derived metabolites (25). Disruptions in aromatic amino acid metabolism are of particular interest, as tryptophan and tyrosine serve as key precursors for neuroactive and immunomodulatory molecules, such as serotonin and kynurenine (26). Consistent with this, our metagenomic analysis revealed perturbations in GBM activity, suggesting that altered microbial metabolism may contribute to immune and neurohumoral disturbances in AKI (3). In addition, microbiota-derived tryptophan metabolites have been shown to regulate host immune responses and epithelial barrier integrity through pathways such as the aryl hydrocarbon receptor (AhR) (27), highlighting a potential mechanistic link between host metabolic alterations and gut microbiota-mediated signaling. Furthermore, several tryptophan-derived uremic toxins, including indoxyl sulfate, have been shown to exert direct nephrotoxic effects by promoting tubular injury, oxidative stress, and fibrosis (28, 29). This provides a biologically plausible connection between the metabolic alterations identified in our study and the progression of renal injury. Among the metabolites identified in our cohort, several exhibited strong diagnostic performance. Notably, maltol (C11918) achieved an AUC of 0.961, comparable to previously reported metabolomic biomarkers for early AKI detection in surgical and septic settings (30, 31). These findings highlight the potential clinical utility of metabolite-based signatures for AKI risk stratification and early prediction.

Metagenomic profiling revealed a significant reduction in α-diversity and major compositional shifts in the gut microbiota of AKI patients. A particularly striking feature was the consistent depletion of key short-chain fatty acid (SCFA) producing commensals, including *Faecalibacterium prausnitzii, Roseburia, Eubacterium rectale* and *Blautia*, a pattern widely recognized as a hallmark of AKI-associated dysbiosis (32–37). This depletion of SCFA-producing taxa is consistent with accumulating evidence from recent studies on the gut-kidney axis, which indicate that reduced SCFA production and concurrent accumulation of uremic toxins represent central mechanisms linking gut microbial dysbiosis to renal dysfunction (13, 14). In addition, microbiota alterations have been associated with impaired intestinal barrier integrity and activation of pro-inflammatory pathways, including TLR4-mediated signaling, thereby contributing to systemic inflammation and kidney injury (13, 17). Recent multi-omics investigations further support the concept that microbial-derived signals play an important role in modulating renal outcomes, and highlight the value of integrative approaches combining microbial and metabolic profiling to better understand disease mechanisms (21). In this context, our findings extend current knowledge by characterizing not only taxonomic alterations but also the functional metabolic potential of the AKI-associated microbiome through GMM and GBM analyses, revealing perturbations in neuroactive metabolic pathways.

These butyrate-producing genera play essential roles in tight-junction reinforcement, epithelial barrier maintenance, and mucosal immune homeostasis, including the support of colonic Treg populations and suppression of NF-κβ-mediated inflammatory pathways (38, 39). In our cohort, their depletion correlated strongly and negatively with pro-inflammatory cytokines (IL-6, IL-8) and renal injury markers (SCr, BUN), providing direct clinical evidence of their protective role. Loss of this “microbial shield” is mechanistically significant: reduced SCFA production compromises epithelial integrity, increases gut permeability, and facilitates translocation of microbial products such as LPS and bacterial DNA, thereby promoting systemic endotoxemia (32, 40). These circulating microbial signals can activate innate immune pathways, including renal TLR4 and other pattern-recognition receptors, amplifying inflammation and accelerating tubular injury, consistent with core predictions of the gut-kidney axis framework (41). This causal link is supported by elegant murine studies demonstrating that fecal microbiota transplantation (FMT) from healthy donors attenuates ischemic AKI, whereas antibiotic depletion of SCFA-producing bacteria abolishes this protective effect (42). Collectively, our findings reinforce the concept that the collapse of SCFA-producing taxa constitutes a critical ecological and immunometabolic inflection point in AKI pathogenesis.

Conversely, although prior studies have highlighted blooms of classic Enterobacteriaceae pathogens such as *Escherichia coli* and *Klebsiella pneumoniae* in AKI, our data indicate a more refined pattern. Among the taxa showing numerical increases, only *Bacteroides uniformis* was consistently enriched across differential abundance, LEfSe, and functional analyses. As a mucin glycan specialist equipped with polysaccharide utilization loci (PULs) such as susD (43, 44), *Bacteroides uniformis* likely expands by exploiting host-derived mucins released during intestinal barrier disruption a mechanism previously observed in inflammatory bowel disease and critical illness (45). Furthermore, its association with isovaleric acid production, a branched-chain fatty acid produced from valine catabolism, further suggests metabolic consequences of this expansion. While BCFAs can serve as alternative substrates, higher levels have been linked to mitochondrial stress and oxidative injury in renal tubules (46). Together, these observations support a model of “metabolic opportunism,” in which a normally commensal species gains advantage in the disease-altered gut environment, reflecting resource-driven ecological restructuring rather than simple pathogen overgrowth (47).

Functional profiling revealed a clear metabolic shift in the AKI gut microbiome. The enrichment of xenobiotic degradation pathways suggests a compensatory microbial response to the accumulation of uremic solutes that the injured kidney cannot efficiently clear (21). Concurrently, the upregulation of amino acid catabolism pathways (e.g., glutamine and cysteine degradation) suggests a transition toward a catabolic microbial state commonly associated with inflammation-driven dysbiosis (48). This metabolic shift may contribute to the production of potentially harmful metabolites, such as ammonia and hydrogen sulfide, which have been implicated in epithelial barrier disruption and systemic toxicity (49, 50). Recent integrative studies further indicate that functional remodeling of the gut microbiome in kidney injury is characterized by enhanced proteolytic fermentation and increased generation of nitrogenous and sulfur-containing metabolites, reinforcing the link between microbial metabolism and host inflammatory burden (51–53). In addition, increased activity in GABA and glutamate synthesis pathways was observed, suggesting enhanced microbial production of neuroactive metabolites. These molecules are increasingly recognized as key mediators of the microbiota-gut-brain axis, with the capacity to influence autonomic regulation and systemic immune responses (35, 54). Such pathways have also been implicated in modulating renal inflammation and stress signaling, highlighting a potential neurohumoral dimension of gut-kidney axis interactions in AKI (55). Emerging evidence suggests that microbiota-derived neuroactive compounds may influence distant organ function through immune-neuroendocrine crosstalk, thereby contributing to systemic inflammatory responses and organ dysfunction in acute disease settings (56).

Co-occurrence network analysis demonstrated that AKI microbial communities exhibit higher connectivity but markedly reduced modularity, indicating an unstable, centralized ecosystem. Ecological theory suggests that such configurations are more vulnerable to perturbations and less resilient to environmental stressors (57). This instability may diminish the resilience of the AKI gut microbiome to antibiotic exposure, infection, or dietary stress, facilitating sustained dysbiosis and systemic inflammation. Similar network-level disruptions have been reported in other inflammatory and metabolic disorders, where reduced modularity is associated with loss of functional redundancy and decreased ecosystem resilience, further supporting the concept of microbiome instability as a hallmark of disease states (58). It is important to interpret these findings within the broader context of AKI research. While previous multi-omics studies have primarily focused on specific etiologies, such as sepsis-associated or drug-induced AKI, the present integrative analysis across a heterogeneous AKI population suggests the presence of conserved microbial-metabolic signatures that may transcend individual triggers. The consistency between our observations and established frameworks of metabolic reprogramming and gut microbiota dysbiosis supports the potential generalizability of these findings (14, 59, 60).

Importantly, these findings should also be interpreted in the context of concurrent systemic metabolic disturbances reflected in baseline clinical characteristics. In the present cohort, AKI patients exhibited not only elevated renal injury markers but also significant increases in liver function indices and fasting glucose levels. Emerging evidence indicates that acute liver dysfunction and stress-induced hyperglycemia can independently reshape gut microbial composition and circulating metabolite profiles through mechanisms involving the gut–liver axis and systemic metabolic stress responses. Hepatic dysfunction has been associated with altered bile acid metabolism, impaired intestinal barrier integrity, and secondary microbial compositional shifts, whereas hyperglycemia may promote pro-inflammatory microbial configurations and disrupt host–microbiome metabolic homeostasis (61, 62). These processes may overlap with, or amplify, the gut microbial dysbiosis and metabolic reprogramming observed in AKI. Therefore, a proportion of the microbial and metabolite alterations identified in this study may reflect combined effects of kidney injury and systemic metabolic perturbations rather than kidney-specific changes alone. This highlights the complexity of multi-organ interactions in AKI and underscores the importance of cautious interpretation. Future studies incorporating stratified analyses and multi-organ functional assessments will be necessary to disentangle the relative contributions of kidney injury, liver dysfunction, and metabolic stress to the observed multi-omics signatures.

However, several limitations should be acknowledged. First, the relatively small sample size and cross-sectional design may limit statistical power and preclude causal inference. In addition, the diagnostic performance of candidate metabolites was evaluated within the discovery cohort without independent external validation. Given the limited sample size, there is a potential risk of overfitting, and the generalizability of these findings may be restricted. Therefore, these results should be interpreted as exploratory and require validation in larger, independent cohorts using more robust modeling strategies. Second, given the heterogeneity of AKI etiologies, the lack of subgroup or stage-stratified analyses may obscure etiology-specific and temporal microbial-metabolic patterns. Third, this study primarily relies on bioinformatic and statistical approaches, including functional prediction and correlation analysis, without experimental validation at the molecular or cellular level. Therefore, the observed associations between gut microbiota, metabolites, and kidney injury should be interpreted with caution and do not establish causality. In addition, although MS/MS spectral matching was applied to improve metabolite annotation confidence (corresponding to MSI Level 2 identification), the absence of validation using authentic chemical standards (MSI Level 1) may still introduce uncertainty in metabolite identification. Furthermore, early tubular injury biomarkers such as kidney injury molecule-1 (KIM-1) and neutrophil gelatinase-associated lipocalin (NGAL) were not assessed, and AKI stage-specific analyses were not performed, which may limit the ability to capture early or stage-dependent metabolic alterations. Recent methodological advances highlight the importance of integrating longitudinal multi-omics designs with experimental validation strategies, such as targeted metabolomics, *in vitro* functional assays, and *in vivo* microbiota-based models, to establish causal relationships and enhance translational relevance (15, 17).

In summary, this study provides an integrative characterization of gut microbiota dysbiosis and metabolic reprogramming in AKI. The findings support a model in which AKI is associated with both structural disruption of microbial communities and functional metabolic reconfiguration, including depletion of beneficial short-chain fatty acid (SCFA)-producing taxa and enrichment of adaptive microbial metabolic pathways. These alterations are closely linked to systemic inflammation, metabolic imbalance, and renal injury. Several plasma metabolites demonstrated promising discriminative potential (e.g., maltol (C11918), AUC = 0.961), and a simplified metabolite panel identified through machine learning-based feature selection further supported their potential relevance. However, given the limited sample size and the absence of external validation, these findings should be interpreted as exploratory and hypothesis-generating. Overall, this study highlights the coordinated interplay between gut microbiota and host metabolism in AKI and provides a framework for future investigations. Further studies incorporating larger, well-stratified cohorts, longitudinal sampling, and experimental validation are warranted to confirm these findings and to clarify causal mechanisms, thereby facilitating clinical translation.

## Data Availability

The datasets presented in this study can be found in online repositories. The names of the repository/repositories and accession number(s) can be found in the article/Supplementary material.

## Footnotes

1
https://www.metaboanalyst.ca

## Supplementary material

Supplementary Table 1 Summary of whole-metagenome shotgun sequencing data quality and filtering statistics for all samples.

Supplementary Table 2 Quantitative features of identified metabolites in HCs, AKI patients, and quality control (QC) samples.
